# Effects of Pooling Samples on the Performance of Classification Algorithms: A Comparative Study

**DOI:** 10.1100/2012/278352

**Published:** 2012-04-30

**Authors:** Kanthida Kusonmano, Michael Netzer, Christian Baumgartner, Matthias Dehmer, Klaus R. Liedl, Armin Graber

**Affiliations:** ^1^Institute for Bioinformatics and Translational Research, UMIT, 6060 Hall in Tyrol, Austria; ^2^Faculty of Chemistry and Pharmacy, Leopold-Franzens-University Innsbruck, 6020 Innsbruck, Austria; ^3^Institute of Electrical and Biomedical Engineering, UMIT, 6060 Hall in Tyrol, Austria; ^4^Novartis Pharmaceuticals Corporation, Oncology Biomarkers and Imaging, One Health Plaza, East Hanover, NJ 07936, USA

## Abstract

A pooling design can be used as a powerful strategy to compensate for limited amounts of samples or high biological variation. In this paper, we perform a comparative study to model and quantify the effects of virtual pooling on the performance of the widely applied classifiers, support vector machines (SVMs), random forest (RF), *k*-nearest neighbors (*k*-NN), penalized logistic regression (PLR), and prediction analysis for microarrays (PAMs). We evaluate a variety of experimental designs using mock omics datasets with varying levels of pool sizes and considering effects from feature selection. Our results show that feature selection significantly improves classifier performance for non-pooled and pooled data. All investigated classifiers yield lower misclassification rates with smaller pool sizes. RF mainly outperforms other investigated algorithms, while accuracy levels are comparable among all the remaining ones. Guidelines are derived to identify an optimal pooling scheme for obtaining adequate predictive power and, hence, to motivate a study design that meets best experimental objectives and budgetary conditions, including time constraints.

## 1. Introduction

High-throughput technologies generate large amounts of data, which allow analysis of a broad range of biomolecules in living organisms [1, 2]. For example, the transcriptome, proteome, and metabolome can be studied by exploiting high-dimensional datasets that comprise RNAs, proteins, and metabolites, respectively. One of the most useful techniques, that have been applied to high-dimensional biological data, is sample pooling. It is a technique where subsets of samples are randomly selected and pooled within each group, and the cardinality of the samples subset is termed pool size. Pooling helps to cut experimental costs and reduces analytical run times; furthermore, it can compensate for limited amounts of samples or can mitigate effects of biological sample variation. Many biological experiments have been performed by pooling individual biological specimens (e.g., [3, 4]). For instance, messenger RNA (mRNA) samples are pooled together before hybridization in a microarray experiment. Instead of employing as many array chips as number of samples *n*, actually required chips are reduced by a factor 1/*p*, where *p* is the pool size.

The effects and efficiency of pooling samples have been statistically investigated in many studies [5–9], which showed that appropriate pooling can provide equivalent power as obtained in comparable studies, where samples of individual subjects are not pooled (i.e., pool size is equal to 1). Thus, it becomes very interesting to study the effects of virtual pooling on high-dimensional classification problems. Recently, this very active research area in bioinformatics has received widespread attention in the biomedical scientific community; primarily, as a result of the recent medical paradigm shift towards personalized medicine. This new strategy reflects an early and ongoing focus on targeted medicines, driven by a rigorous pathways approach to drug and biomarker discovery, which incorporates the qualification of biomarkers and their translation into companion diagnostics in the codevelopment process. The discovery and qualification of biomarkers as well as assay development, validation, and commercialization are empowered by an unprecedented evolution and emergence of exciting new molecular technologies including high-throughput “omics” microarrays, next-generation sequencing, functional imaging, and evolving nanotechnologies. Classification methods have been commonly employed to discover and validate sets of biomarkers in system-wide biomedical studies that fulfil predefined performance metrics, demonstrate clinical utility, and meet technical, practical clinical, and business-related expectations, which permit pursuing the development and commercialization of a clinical assay. Those studies frequently aid the prognostic and diagnostic assessment, and the predictive comparison of treatments of diseases such as cancer [10, 11], liver [12], or neurodegenerative diseases [13].

Recently, a study has been published investigating effects induced by pooling data [14]; however, this analysis did not explicitly include feature selection, which is a frequently used analysis step in high-dimensional classification problems. Feature selection is applied prior to the classification process for reducing noise features and selecting key features, which in general leads to a better discrimination by classification methods.

In the current work, we investigate the impact of pooling biological samples on classification algorithms in combination with feature selection. The data employed in our study are systematically synthesized with various numbers of markers and different human and animal (e.g., mice or rats) data scenarios. The data of human scenario mimick real-life experiments with larger sample size and higher biological variance comparing to animal scenario. A comparative study on the performance of commonly used classifiers in nonpooled and pooled data is performed. We apply supervised machine learning where predictive functions are deduced from training data. We focus on five important classifiers, support vector machines (SVMs) using linear and radial kernels, random forest (RF), *k*-nearest neighbors (*k*-NNs), penalized logistic regression (PLR), and prediction analysis for microarrays (PAMs).

Technical preliminaries of pooling samples, investigated classification algorithms, and feature selection are described in the next section. The materials and methods of data simulation and analysis framework are then explained. As results of this study, first we report the benefits of feature selection on both non-pooled and pooled datasets. Then, the effects of pooling data are presented. This comparative evaluation depicts the performance of classifiers on datasets of individual and pooled samples with several pool sizes. We also provide a comparison of human and animal scenarios, denoting the simulation of datasets that exemplify data characteristics typically observed in human studies and animal experiments. These results are discussed according to properties of data and classification algorithms. We conclude the work by deducing guidelines for the selection of classification algorithms and pool sizes that allow researchers to identify a study design that meets best their experimental objectives and budgetary conditions, including time constraints.

The main contribution of our paper is as follows: a thoroughly chosen experimental design, which combines an applicable pool size with a proper classification algorithm, allows constructing predictive models with performance characteristics comparable to models resulting from regular non-pooled strategies. These pooling designs can be primarily applied in biomarker discovery studies to build classification models to predict the class of future non-pooled subjects. Depending on the application and clinical utility of respective classifiers, such predictions might relate to the diagnosis and prognosis of disease, or the prediction of treatment success for individual patients.

## 2. Technical Preliminaries

### 2.1. Pooling Samples

For a general high-throughput experimental setup, let *n* denote the number of samples, and *m* represent the number of pooled samples or performed experiments (e.g., microarray chip or mass spectrometry runs). Thus, *m* is equal to *n* in non-pooled experiments. The observed value of a feature *f* in sample *i* is denoted by *z*_*i*_. We assume for each feature *f* (e.g., gene expression or metabolite concentration) that *z*_1_,…, *z*_*n*_ are independent and identically distributed random variables with mean *μ*_*f*_ and biological variance *σ* ^2^. By considering the technical variance *ε*_*k*_ ~ *N*(0, *σ*_*ε*_^2^) to account for experimental variability, the experimental measurements can be represented as

(1) $$\begin{array}{c} y_{i}=z_{i}+\varepsilon_{i}. \end{array}$$

The biological and technical variations are independent. Assuming that each individual contributes equally to the pool, the pooled value *z*′ is the average of *p* individuals

(2) $$\begin{array}{c} z^{\prime }=\frac{1}{p}\overset{p}{\underset{i=1}{\sum }}z_{i}, \end{array}$$

where *p* refers to the pool size. In this study, we consider designs where *n* = *pm* and *m* is the number of pooled samples. The measured values of pooled samples *y*_1_′,…, *y*_*m*_′ can be represented as

(3) $$\begin{array}{c} y_{k}^{\prime }\;=z_{k}^{\prime }+\varepsilon_{k}, \end{array}$$

where *ε*_*k*_ ~ *N*(0, *σ*_*ε*_^2^) as in (1). The biological variance of pooled sample is then reduced to *σ* ^2^/*p* [14, 15].

### 2.2. Classification Algorithms

The general procedure in classification is to train a classifier on labeled training samples and to classify future unlabeled samples employing the trained model [16, 17]. Let *x* be a data set of *a* variables {*f*_1_,…, *f*_*a*_}, called features and *c*_*i*_ be a class variable. Then a classifier is a function *f* : *f*_1_ × ⋯×*f*_*a*_ → *c*_*i*_.

SVMs can be explained by four basic concepts: (i) the separating hyperplane, (ii) the maximum margin hyperplane, (iii) the soft margin, and (iv) the kernel function [18, 19]. SVMs construct a separating hyperplane which gives the largest separation margin between two classes. Soft margin allows some errors occur between the separation and a kernel function maps data into higher dimensional data space allowing the linear separation in nonlinear classification problems [12, 20].

RF is an ensemble-based machine learning method, which relies on the aggregation of results from several individual decision trees [21]. Each tree in the procedure is constructed by bagging data from the original dataset. A number of features are randomly selected to build a tree. The predicted class for each sample is then assumed to be the class that obtained the majority vote based on all trees.

*k*-NN is an instance-based learning method [16]. Giving a new query point (i.e., sample) *x*, *k*-NN finds *k* points in a training set, which are closest in distance to *x*. The class of *x* is determined by majority voting of *k* nearest neighbors using, for example, the Euclidean distance as metric.

PLR combines the logistic regression criterion with a penalization of the *L*_2_-norm of the coefficients which enables a stable fit, even with a large number of features [22]. It performs similarly to SVMs, but in addition provides an estimate of the underlying probability [23].

PAM classifies samples based on the method of *nearest shrunken centroids*. The method assigns an unknown sample to the class whose centroid is closest (i.e., smallest squared distance). The centroids are *shrunken* by moving class centroids toward the overall centroids after standardizing by the within-class standard deviation for each feature. If the difference between the centroids of all classes and the overall centroid is smaller than a user-defined threshold for a feature, the feature is eliminated [24].

### 2.3. Feature Selection

The performance of classifiers strongly depends on properties of the feature set, such as which information is irrelevant or redundant. Feature selection uses different measures to select relevant features and is an important first step in building diagnostic, prognostic and predictive models [25]. Methods for feature selection can be classified into filter, wrapper, and embedded methods [26]. Wrappers use estimates of discriminatory performance (e.g., accuracy) provided by machine learning approaches to evaluate feature subsets. Similar to wrappers, embedded methods integrate with classifiers but take into account search strategies that require less computational power. Filter methods rank features based on their ability to distinguish between predefined classes and are independent of the classification algorithm and easier to interpret. In this paper, we apply statistical hypothesis testing, the Student's *t*-test, which is commonly used in bioinformatics.

## 3. Materials and Methods

### 3.1. Datasets

The mock datasets have been generated by simplicity mimicking various biological scenarios. Let *n* be the number of samples and *a* denote the number of features. A sample consists of features {*f*_1_,…, *f*_*a*_}, which represent, for example, gene expressions or metabolite concentrations in a biological context. The dataset can be described as a set of samples *D* = {(*x*_*i*_, *c*_*i*_) | *x*_*i*_ ∈ *X*, *c*_*i*_ ∈ *C*}, where *X* is a set of samples, *x*_1_,…, *x*_*n*_, and *C* is a set of class labels. The data are balanced and dichotomous with a set of class labels *C* = {control, case}. For each feature, samples among each class are assumed to follow a Gaussian distribution, which is denoted as follows:

(4) $$\begin{array}{c} X_{\text{control}}\sim N\left(0,\sigma^{2}\right),\\ X_{\text{case}}\sim N\left(\gamma ,\sigma^{2}\right). \end{array}$$

  *σ* ^2^ is considered as biological variance. *γ* denotes the relative mean difference between two groups. We define a discriminator (i.e., biomarker in biological context) as a feature with *γ* ≠ 0. In this study, *γ* is randomly chosen from uniform distribution *U*(0.3,0.4) [27]. Thus, the value of *γ* in the case group of a nonmarker is 0, otherwise it is greater than 0. In addition, the technical variance is taken into account as *ε*_*i*_ ~ *N*(0, *σ*_*ε*_^2^ = 0.2^2^) according to (1). The numbers of markers are varied from 1 to 10.

From the data properties described above, we consider two simplified scenarios of data set characteristics. Scenario 1 is defined with *a* = 1000 and *n* = 90 per class. The biological variance *σ* ^2^ has the value of 0.2. These assumptions are used to simulate human data set characteristics. Scenario 2 is defined with *a* = 1000, *n* = 30 per class, and *σ* ^2^ = 0.1. The later assumptions are used to imitate animal (e.g., mice and rats) data set characteristics. Human biomarker discovery studies are generally designed and executed with a higher number of samples than animal experiments. On the other hand, the variability in animal experiment is smaller than in human settings according to in-bred and genetic homogeneity of study subject populations as well as better means to standardize and control experimental conditions [28].

### 3.2. Pooling Data Simulation

Let *p* be the number of samples that are pooled. In this study, we set *p* = 2, 3, and 5, that *n* = *pm*, where *m* is the number of pooled samples. Most measurements of pools were reported to be similar to averages of individuals comprising the pool [8, 15]. Thus, in this study, each pooled sample was obtained by averaging *p* samples. For *p* = 2, 3, and 5, the pooling datasets are in sizes of 90, 60, and 36 instances in the human scenario of total 180 samples and in size of 30, 20, and 12 instances in the animal scenario of total 60 samples, respectively. In order to mimic a real-life experiment, in which the pooling is done before the samples are analyzed, the simulated data were transformed by performing exponential function to the basis *e* prior to pooling [29]. Then the pooled data were transformed back into the natural log scale. The new value of derived pooled samples *y*′ can be represented as

(5) $$\begin{array}{c} y^{\prime }=\log_{e}\left(\frac{1}{p}\overset{p}{\underset{i=1}{\sum }}e^{z_{i}}\right)+\varepsilon_{k}, \end{array}$$

where *z*_*i*_ denotes the value of each individual sample and *ε*_*k*_ denotes technical errors, *ε*_*k*_ ~ *N*(0, *σ*_*ε*_^2^ = 0.2^2^) of a pooled experiment as applied from (2) and (3). Note that each value *y*′ is calculated for each feature.

### 3.3. Classification, Feature Selection, and Model Evaluation

The discriminatory ability of popular classifiers, which are SVMs using both linear and radial kernels, RF, *k*-NN, PLR, and PAM are compared based on synthetic data. Feature selection by using statistical *t*-test is included. The features are ranked according to the *t*-statistics and the top 10, 100, and 1000 features are selected for classification. The implementation of the R package classification for microarrays (CMAs) [30] was used for feature selection, classification and model evaluation. In this work, for model evaluation, we did not perform common-applied *k*-fold cross-validation (CV), which subdivides data into *k* partitions and each turn uses one partition as test set and the remainder as training set. This is specific to the pooling problem since in real life the constructed classifier only utilize future individual sample for class prediction. The training sets can be pooled since the classes of subjects are already known. However, new subjects cannot be pooled for testing as they might belong to different classes. Thus, the CV or even other model evaluation method, for example, bootstrap cannot be applied in the pooling approach as the test set cannot consist of pooled samples in real use. Consequently, we used separate training and test sets for model evaluation. Classifier construction utilizes a training set and then model validation is performed by using a test set. The test set comprised 450 individual samples and the average misclassification rates from each test sample was obtained. The pipeline from data simulation to model evaluation was repeated 300 times. The selected number of test samples and the number of replications were found to give a small variance and stable results in our setting, respectively.

Feature selection was performed for each training set. A number of top ranked features (10, 100, and 1000) were selected based on a training data. The selected ranked features were then utilized in the test set for model estimation.

Parameter tuning for every classifier was performed using internal 3-fold CV with customized grids [30] on the training set. The number of folds was found to have no significant effect on classifier performance. By applying a 3-fold CV strategy, the training set was subdivided into three equal partitions where each one took turns and consecutively was used for model validation, and the remainder for training. Finally, the optimal parameters were derived from the CV. By performing CV, soft margin values (*c*  = 0.1, 1, 5, 10, 50, 100, 500) were tuned for SVMs both linear and radial kernel. The gamma values (0.25, 0.50, 1.00, 2.00, and 4.00) were determined for radial kernel. For RF, numbers of randomly sampled features (4, 8, 16, 32, and 64 considered based on squared root of total features which is 1000) were adjusted and the number of trees was set to 1000. A *k* value (1 to number of top ranked features) was selected for *k*-NN. The lambda values (0.0625, 0.1250, 0.2500, 0.50, 1.00, 2.00, 4.00, 8.00, and 16.00) were tuned for PLR. The thresholds for deltas were searched among (0.1, 0.25, 0.5, 1, 2, and 5) in PAM.

## 4. Results and Discussion

Five well-known classifiers, comprising SVMs using both linear and radial kernels, RF, *k*-NN, PLR, and PAM, were selected to investigate discriminatory performance for (i) different number of top ranked features, (ii) different pooling sizes including different numbers of virtual discriminators (i.e., biomarkers in biological context), and (iii) human and animal (e.g., mice or rats) scenarios.

### 4.1. Effects of Feature Selection

In this study, we used the Student's *t*-test, the most popular statistical test to filter genes [31], for feature selection. Filter methods have the advantages of classifier independence, lower computational complexity, and they provide ranking and prioritization of features which are important for biological contextualization and interpretation [26].

Results (based on the human scenario) demonstrate that the examined classifiers generally show significantly smaller misclassification rates (using the Wilcoxon rank sum test) when employing feature selection in both individual and pooled data, compared to runs without feature selection (Table 1). This observation can be explained by the ability of feature selection to reduce noise and to avoid model overfitting. The findings are in concordance with several other studies showing that feature selection methods yield better discriminatory power of classification algorithms (e.g., [12, 27]). However, PAM performs better without feature selection when compared with the parameter setting where the 100 top-ranked features are selected in our datasets. This may be an effect of internal feature selection and the optimal parameter delta from parameter tuning, which shrinks the standardized centroid for each class in this particular algorithm [24].

### 4.2. Effects of Pooling Samples

In order to investigate the effects of pooling samples on classification algorithms, datasets of different numbers of pooled samples were mimicked (see Section 3). The evaluation was based on human scenario and 100 top-ranked features using the *t*-test for feature selection.

**Figure 1:**
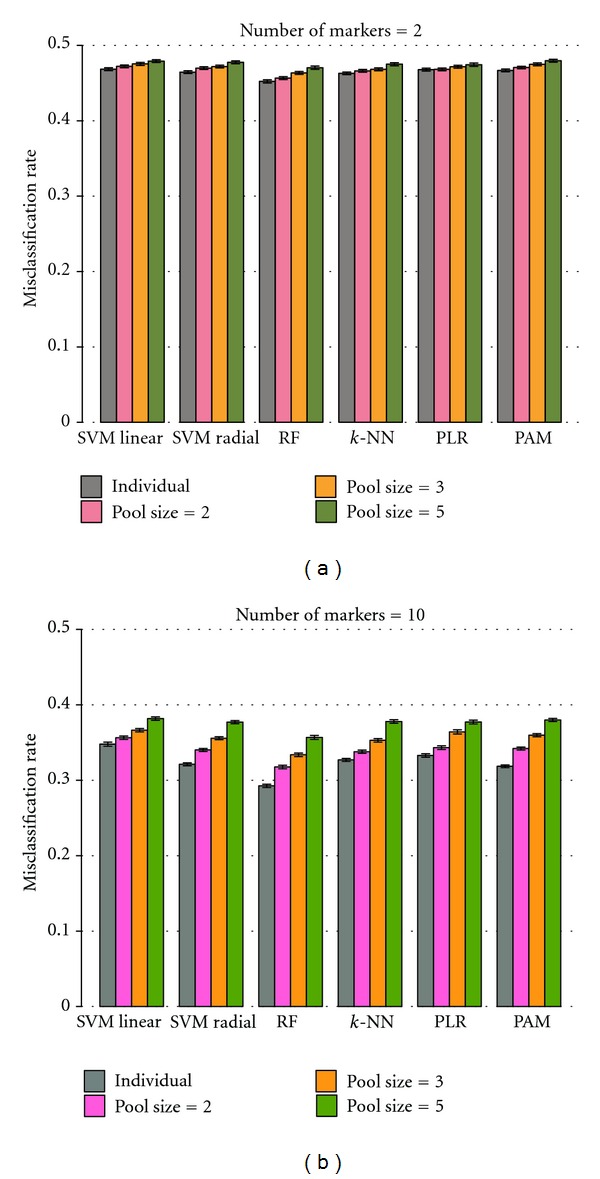
Performance of classifiers on individual samples and various pool sizes. A comparison of classifiers performances for datasets of individual samples and pool sizes of 2, 3, and 5 are shown. Misclassification rates rise with larger pool sizes. (a) and (b) show the comparison when numbers of markers are 2 and 10, respectively. The height of bars indicates 95% confidence interval from 300 replications.

Misclassification rates obtained by the six classifiers were investigated for individual subjects and pooled samples (pool sizes of 2, 3, and 5). The results show that the misclassification rates increase with larger pool sizes (Figure 1), which is in accordance with the study of Telaar et al. [14]. This characteristic can be observed with both small and larger numbers of markers in datasets. Although pooling helps to decrease variances of biological samples, the sample size is reduced when samples are pooled [15] which can degrade the discriminatory ability of classifiers. In addition, the increase of misclassification rates with raising pool sizes follows a linear pattern. The difference among the performance of classifiers is larger for higher numbers of markers than for small numbers of markers in the data.

**Figure 2:**
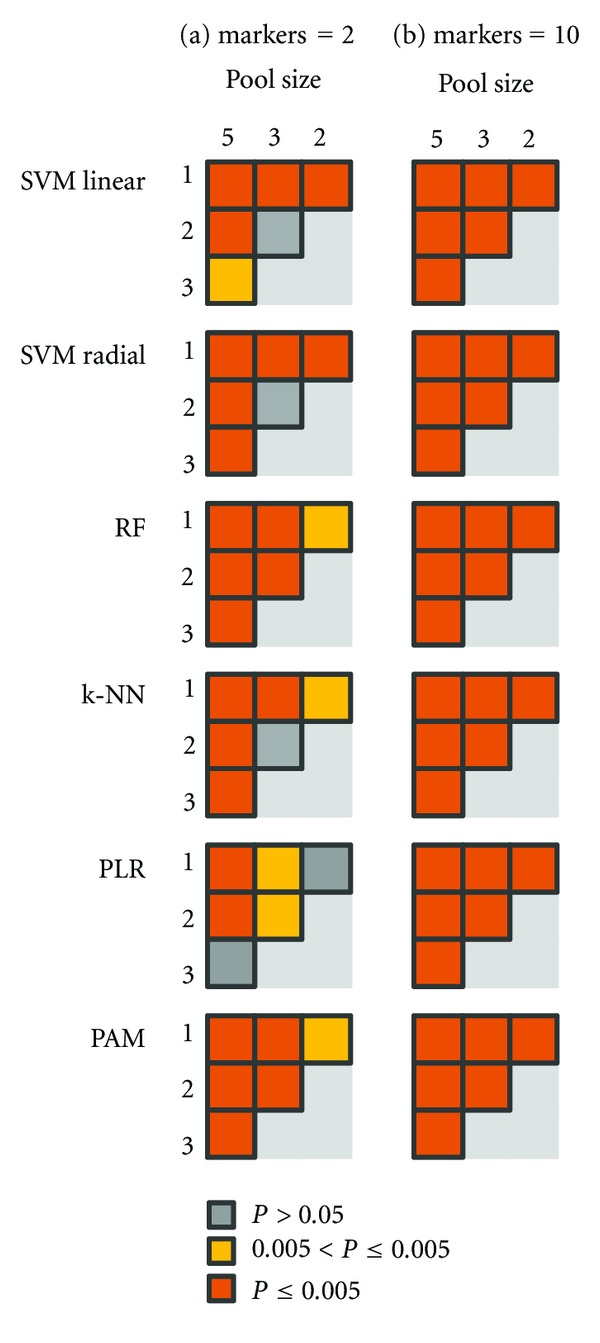
Statistically significant matrices of classifiers performances among various pool sizes datasets. Statistically significant differences of performances of classifiers among different pool sizes of 1 (individual), 2, 3, and 5 are shown using Wilcoxon rank sum test, respectively. (a) and (b) show the matrices when numbers of synthesized markers are 2 and 10, respectively. Each square represents a comparison between two pool sizes datasets. The colors indicate the level of significance.

Significant differences in the performance of classifiers between individual subjects and various pool sizes become apparent from the Wilcoxon rank sum test (Figure 2). In datasets with large number of markers, the performances of classifiers show significant differences in every pair of pool size (Figure 2(b)). On the other hand, in the datasets with small numbers of markers, there is no statistical significant difference (*P* > 0.05) between some pairs of pool sizes (Figure 2(a)). For example, there is no statistically difference between individual sample and pool size = 2 and between pool size = 3 and pool size = 5 in PLR. For SVMs with both linear and radial kernels, performances of classifiers do not show statistical differences at pool sizes of 2 and 3, respectively. These results could motivate the use of classifiers with different pool sizes in cases where the data is noisy and only a small number of markers are expected.

**Figure 3:**
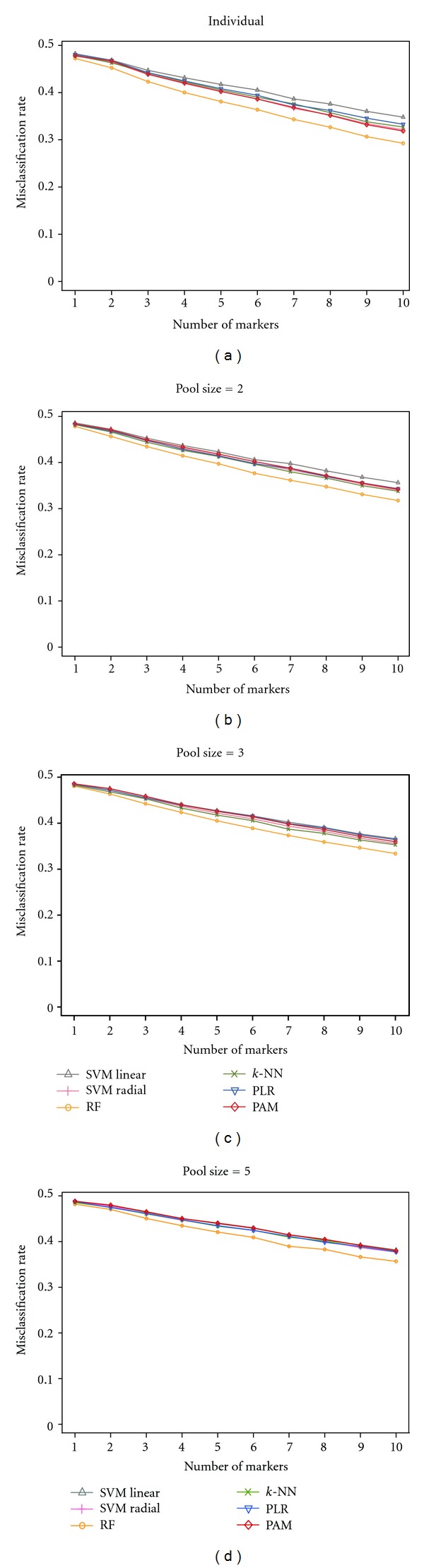
The performance of classifiers for various numbers of markers based on non-pooled and pooled data. Misclassification rates of classifiers are shown when the number of markers is increasing from 1 to 10. (a) shows the performance of classifiers on dataset of individual samples (90 controls and 90 cases). (b)–(d) show the performance of classifiers on pooled datasets when pool sizes are 2, 3, and 5, respectively.

In order to gain further insight on the performance of different classifiers, the misclassification rate of classifiers with different number of markers from 1 to 10 was investigated (Figure 3). RF outperforms other classifiers for every pool size (2, 3, and 5) in our settings (with 100 top-ranked features). For other classifiers, the performance-ranked order slightly differs, depending on the pool size. SVM with linear kernel does not perform as well as SVM with radial kernel in our settings. The kernel function helps to map data into higher dimension space. This could allow the linear hyperplane providing better separation between data points of two classes. The performance variation of classifiers is greater for individual and small pool sizes than for larger pool sizes.

The RF classifier demonstrates a good predictive performance even when most predictive features are noisy and turns out to be robust against overfitting. In earlier studies, it was also reported to produce favorable results [32, 33]. In addition, ensemble methods like RF are generally well-suited for reducing the total expected error [34].

Also performance trends of classifiers with increasing numbers of markers are demonstrated in Figure 3. The higher the number of markers, the better the classification performance [20]. This trend is apparent with any number of pooled data.

### 4.3. Results of the Mimicked Animal Scenario

**Figure 4:**
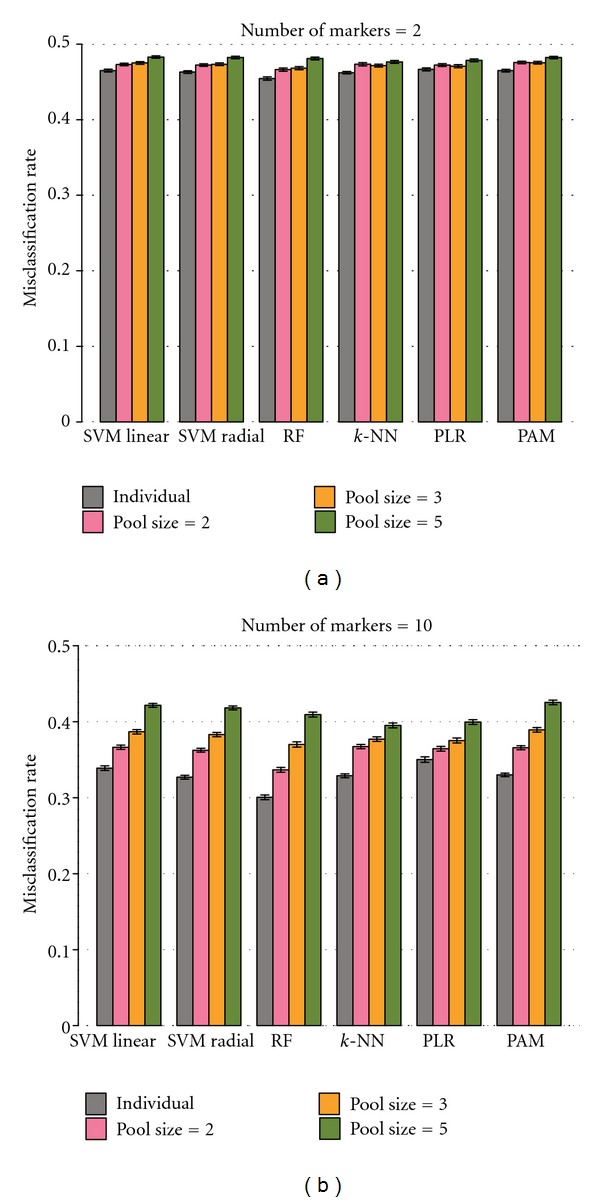
Performance of classifiers based on the animal scenario. Performance of classifiers for datasets of individual samples and pool sizes of 2, 3, and 5 are compared. Misclassification rates rise with larger pool sizes as the human scenario. (a) and (b) show the comparison when numbers of markers are 2 and 10, respectively. The height of bars indicates 95% confidence interval from 300 replications.

To provide a real-life scenario, we mimicked datasets of human studies and animal (in this case mice or rats) experiments. The animal datasets were simulated with a smaller sample size and smaller variance compared to the human scenario (see Section 3), reflecting properties of real-world data [28, 35]. For instance, mice experiments are generally conducted with smaller sample sizes. The variability in mice is smaller than in human settings due to in-bred and genetic homogeneity of populations as well as means to standardize and control experimental conditions (e.g., dietary control, time of sample collection). The effects of pooling samples in the animal scenario are shown in Figure 4.

**Figure 5:**
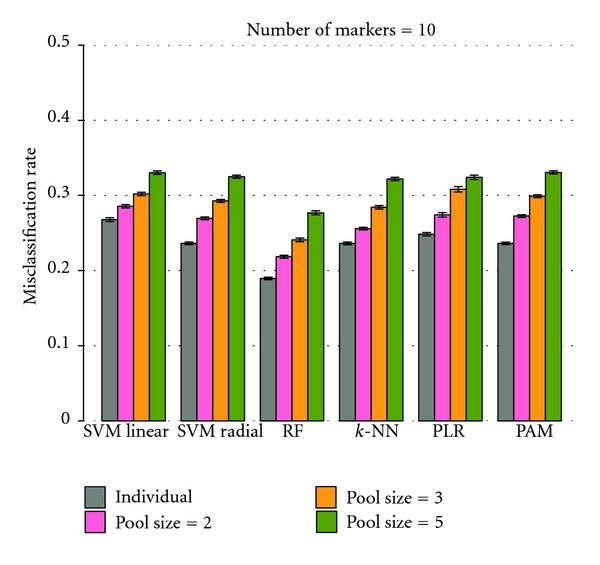
Performance of classifiers based on the animal scenario (sample size = 180). Performance of classifiers for datasets of individual samples and pool sizes of 2, 3, and 5 are compared when numbers of markers = 10 and sample size = 90 samples per class as performed in human scenario. The height of bars indicates 95% confidence interval from 300 replications.

In general, the trends of the animal study simulations (Figure 4) are similar to the human scenario (Figure 1), where a larger pool size causes higher error rates for classifiers. The differences between classifier performances are also larger for bigger numbers of mocked markers in datasets. However, the classifiers produce increased misclassification rates compared to the human scenario despite the lower variance in the animal datasets. The lower variability is compromised by the effect of the sample size. We have investigated the performance of classifiers in the animal study scenario with the same sample size as in the human setting. As expected, the classifiers in the animal scenario outperform the ones in the human setting (Figure 5).

## 5. Conclusions

In this work, we provide a systematic evaluation of pooling designs on the discriminating ability of classifiers. The performance of SVMs, RF, *k*-NN, PLR, and PAM was studied on mock datasets. The results highlight that pooling strategies generally lead to higher error rates of classifiers. Misclassification rates are likely to increase with pool sizes in a linear pattern, not exponentially. Moreover, with datasets having small number of makers, there is no statistically significant difference of the performance of classifiers between some pairs of pool sizes. Although being inferior to non-pooling design, these results suggest the consideration of pooling strategies for “omics” biomedical studies; especially, if there are budgetary or time constraints that do not permit the analytical execution of individual sample runs (e.g., LC/MS-MS). Furthermore, a staged approach might also be considered where first a pooling design is used for global profiling of biomarkers in high-dimensional datasets and subsequent model building, followed by qualification steps where individual samples are analyzed and only a subset of biomolecules is targeted for analysis. This comparative study motivates scientists to consider and balance pros and cons of various designs prior to the execution of biomarker discovery studies. Thus, scientists are encouraged to make an informed decision to leverage pooling designs as a valid strategy to compensate for limited amounts of samples or high biological variation, or as a remedy to improve analytical cost and time efficiency.

In this study, we applied various classifiers with and without feature selection, and systematically explored the effect of relevant parameters on their performance. In general, all considered designs aim at discovering a subset of features via an algorithm that is subsequently used to predict future outcome, such as the disease status. Our results show that RF mainly outperforms SVMs, *k*-NN, PLR, and PAM in our settings, while the latter provide comparable accuracy among themselves. SVMs perform better with a radial kernel compared to a linear one. We strongly recommend conducting feature selection prior to classification. It aids in picking important features and reducing noise, which in turn yields better performance of classification algorithms. The results highlight the importance of applying feature selection and pooling design according to the individual properties of the classification algorithms. As a consequence of the selected data properties in the human and animal study scenarios, sample size influences and compromises the performance of classifiers more than variance of the data in our setting. Therefore, even though data of the animal scenario has lower variance in this study, the classifiers do not perform better than in human datasets.

In future studies, we want to include skewed class distributions and correlations between features in our mock datasets and explore the effect of these properties as well as unbalanced study group sample sizes on the performance of classifiers.

## Figures and Tables

**Table 1:** Comparison of classification performance with different numbers of top-ranked features.

	Misclassification rate		
Classifiers	Top 10	Top 100	Top 1000
Individual			

SVM with linear kernel	0.2191**	0.3479**	0.3757
SVM with radial kernel	0.2178**	0.3211**	0.3712
RF	0.2436**	0.2926**	0.2975
*k*-NN	0.2515**	0.3270**	0.4354
PLR	0.2160**	0.3329**	0.3761
PAM	0.2096**	0.3185**	0.2310

Pool size = 5			
SVM with linear kernel	0.3229**	0.3817**	0.4131
SVM with radial kernel	0.3167**	0.3771**	0.4571
RF	0.3272**	0.3568**	0.3841
*k*-NN	0.3133**	0.3779**	0.4720
PLR	0.3113**	0.3772**	0.3983
PAM	0.3005**	0.3799*	0.3681

Classification performance is presented for different number of top-ranked features. The dataset contains a total of 1000 features and 90 samples per class with 10 markers. Top 1000 features denote no feature selection. The table shows results using individual samples and illustrates results derived by means of a pooled dataset when pool size is 5, respectively. Significance levels *P < 0.05 and **P ≪ 0.05 indicate comparisons where no feature selection is performed by using the Wilcoxon rank sum test.
